# Comment on: homozygous variant p. Arg90His in *NCF1* is associated with early-onset interferonopathy: a case report

**DOI:** 10.1186/s12969-021-00612-3

**Published:** 2021-08-16

**Authors:** George N. Goulielmos, Maria I. Zervou, Elias Eliopoulos

**Affiliations:** 1 Section of Molecular Pathology and Human Genetics, Department of Internal Medicine, School of Medicine, University of Crete, Heraklion, Greece; 2 Department of Internal Medicine, University Hospital of Heraklion, Heraklion, Greece; 3 Laboratory of Genetics, Department of Biotechnology, Agricultural University of Athens, Athens, Greece

Dear Editor,

We read with great interest in Pediatric Rheumatology the article by Schnappauf et al. [1] referred to the association of the homozygous variant p. Arg90His in *NCF1* gene with early-onset interferonopathy. Whilst heterozygosity for the rare rs201802880 p.Arg90His variant of *NCF1* had been associated previously with susceptibility to systemic lupus erythematosus (SLE), rheumatoid arthritis (RA) and Sjögren’s syndrome in adult patients [2], the authors nicely showed, through exome sequencing, the association of the homozygous Arg90His variant with interferonopathy appearing features of autoinflammation and autoimmunity, in a pediatric 5-year old female patient of Indian ancestry [1]. Gene expression analysis was conducted in peripheral blood by an elegant way and an interferon gene expression signature was detected, which was further supported by cytokine analyses of supernatants of cultured patient’s cells. Altogether, these findings suggested that the inflammatory disease developed in the patient was at least in part mediated by type I interferons. Interferonopathies are a group of autoinflammatory diseases characterized by excessive activation of type I interferon that leads to disturbances in immune function. Neutrophil cytosolic factor 1 (NCF1) is an essential component of the gene family encoding NOX2 that represents the phagocytic NADPH oxidase isoform complex, which is an enzyme response for one-electron reduction of molecular oxygen to superoxide [3]. The rs201802880 variant of *NCF1*, altering an arginine to histidine in a PX domain of the NCF1 protein, in humans leads to reduction-of-function of NADPH oxidase [4].

Prompted by the findings of Schnappauf et al. [1] and the fact that rs201802880 is a shared genetic factor involved in the development of various autoimmune diseases, we attempted to elucidate further the functional significance of the Arg90His variant by using a structural biology approach. The role of the highly conserved Arg90 present in a pocket of the p47^phox^ PX Domain has been shown in the past to be the direct involvement in recognition of the polar heads of phosphoinositides [5]. By using the structure of the PX domain of p47phox (PDB code 1KQ6 and [6]) that exhibits a phosphoinositide-binding activity that is normally suppressed by interacting intramolecularly with the C-terminal SH3 domain we are showing here that the substitution of Arg90 (Fig. 1 A) to His (Fig. 1B) not only eliminates the direct electrostatic interactions of the p47^phox^ domain with the phosphate groups (shift from 2.4 to 6 A shown with dashed lines) but also weakens the positive charge distribution on the molecular surface. In addition, the shorter His side chain creates an empty volume between the histidine imidazole group and the phosphate at a distance of 6 A causing either further conformational changes or rehydration of the molecular surface both leading to loss of the p47^phox^ PX Domain to phospoinositide interaction. This is in agreement with the multidiscipline studies by Karathanassis et al. [7] and Ueyama et al. [8] showing respectively that the Arg90Ala and Arg90Lys mutations on the p47^phox^ PX Domain decrease membrane affinities, resulting in longer membrane residence time due to disruption of their interaction with the cognate phospholipid ligand. The loss of the domain’s capacity to bind PI(3,4)P_2_ shows significant loss of translocation to the plasma membrane [5].

**Fig. 1:**
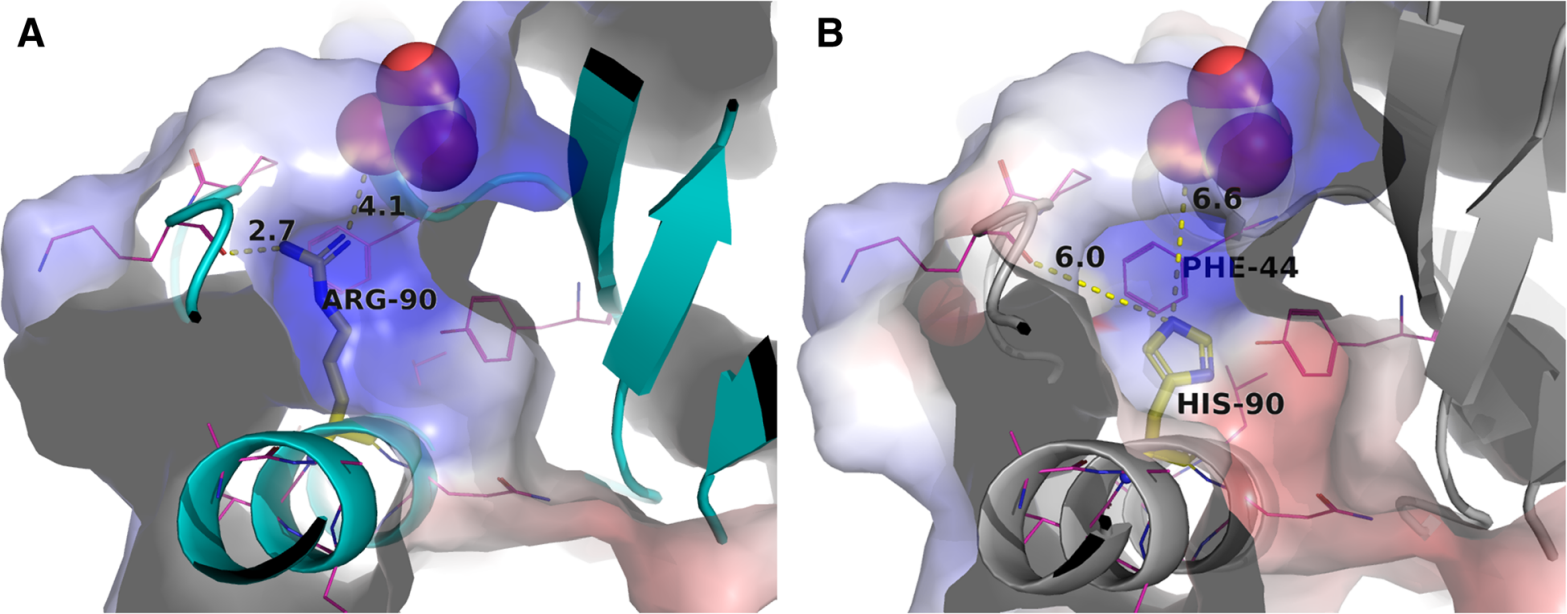
Representation of the phosphate binding pocket of NCF1 p47^phox^ domain with the electrostatic molecular surface. “A” for the native structure an “B” for the Arg90His mutant. The electrostatic surface is shown in color varying from blue (positive) to red (negative). Protein structural elements involved are shown as ribbons, Arg90 and His90 as sticks with the rest of the side chains as lines. The phosphate is shown in space fill representation. The electrostatic surface potential of the models was calculated by the Adaptive Poisson-Boltzmann Solver (APBS) using the PyMOL plug-in with the default parameter settings. The molecular graphics program PyMOL [9] was used to analyze the changes caused by the mutation and the molecular surface charge distribution and to display the results

To our knowledge, this is the first study to evaluate the structural significance of the rs201802880 SNP causing the Arg90His mutation on the p47^phox^ PX Domain. We conclude that this SNP modifies the function of the p47^phox^ PX cytosolic subunit of neutrophil NADPH oxidase leading to affinity reduction to PtdIns(3,4)P_2_ caused by the loss of specific phosphoinositide headgroup interactions and affecting the p47^phox^ translocation to the plasma membrane. This information would help to further interpret the findings of Schnappauf et al. [1] from the structural-functional point of view.

## Data Availability

All data generated during this study are included in this article.
